# Resin Composites with Anti-Biofouling Zwitterionic Polymer and Silica/Zirconia Filler for Digital Light Processing (DLP) of Dental Protheses

**DOI:** 10.3390/ma18153677

**Published:** 2025-08-05

**Authors:** Yun-Hee Lee, Jae-Min Jung, Gyu-Nam Kim, Young-Hag Koh

**Affiliations:** 1 School of Biomedical Engineering, Korea University, Seoul 02841, Republic of Korea; yunheeunique@gmail.com (Y.-H.L.); optima3000@daum.net (J.-M.J.); gyunamkd@korea.ac.kr (G.-N.K.); 2 Interdisciplinary Program in Precision Public Health, Korea University, Seoul 02841, Republic of Korea

**Keywords:** anti-biofouling, biofilm, MPC, silica-zirconia filler, digital light processing

## Abstract

This study aimed to develop an innovative resin composite with anti-biofouling properties, tailored to prosthesis fabrication in dentistry using a digital light processing (DLP) 3D-printing technique. The resin composite was formulated using a blend of dental monomers, with the integration of 2-methacryloyloxylethyl phosphorylcholine (MPC) with anti-biofouling behavior and γ-MPS-treated silica-zirconia powder for simultaneous mechanical reinforcement. The overall characterization of the resin composite was carried out using various contents of MPC incorporated into the resin (0–7 wt%) for examining the rheological behavior, photopolymerization, flexural strength/modulus, microstructure and anti-biofouling efficiency. The resin composite demonstrated a significant reduction in bacterial adhesion (97.4% for *E. coli* and 86.5% for *S. aureus*) and protein adsorption (reduced OD value from 1.3 ± 0.4 to 0.8 ± 0.2) with 7 wt% of MPC incorporation, without interfering with photopolymerization to demonstrate potential suitability for 3D printing without issues (*p* < 0.01, and *p* < 0.05, respectively). The incorporation and optimization of γ-MPS-treated silica-zirconia powder (10–40 vol%) enhanced mechanical properties, leading to a reasonable flexural strength (103.4 ± 6.1 MPa) and a flexural modulus (4.3 ± 0.4 GPa) at 30 vol% (*n* = 6). However, a further increase to 40 vol% resulted in a reduction in flexural strength and modulus; nevertheless, the results were above ISO 10477 standards for dental materials.

## 1. Introduction

The advent of advanced 3D-printing technologies, particularly digital light processing (DLP), has significantly transformed the fabrication of dental prostheses, owing to its distinctive advantages [1,2]. The layer-by-layer additive manufacturing process of DLP enables the production of highly complex structures, which are challenging to achieve with traditional manufacturing techniques [3,4,5,6]. This capability of the DLP process is especially advantageous for the fabrication of dental prostheses, for which customization and adaptability for individuals are critical [7,8]. Furthermore, DLP enables the precise and cost-effective production of temporary prostheses employing photocurable polymeric resins, which undergo solidification rapidly upon exposure to ultraviolet (UV) irradiation [9,10]. The ability of DLP to produce highly accurate, functional, and biocompatible dental prostheses in an efficient and cost-effective manner makes it a powerful manufacturing technique in modern restorative dentistry, satisfying both clinical standards and patient preferences [11,12].

Despite the remarkable precision and versatility of DLP in creating high-quality dental devices, there is an ongoing challenge in the field of dental prostheses, as biofilms can form on the surfaces of those prostheses [13]. A biofilm is a three-dimensionally deposited layer of microbial communities. It is initially triggered by the attachment of bacteria on the surface of a substrate in the oral environment [14,15]. The role of bacteria as a major contributor to the generation of extracellular polymeric substance (EPSs) [16,17] is particularly problematic since it accelerates additional bacterial adhesion on the surface of a substrate. This behavior results in various oral health issues, including microbial-influenced corrosion [18,19], oral diseases such as secondary caries and gingivitis [20], and the failure of implantation in the human body [21,22,23].

Considering the inevitability of biofilm formation in the oral environment, where microbial colonization is a continuous process, there is an urgent need for the development of novel resin composites with antimicrobial properties. In biomedical and dental applications, biofouling, which refers to the undesirable accumulation of proteins, bacteria, and other microorganisms on surfaces, represents a considerable obstacle [24,25]. To mitigate these issues, 2-methacyloyloxyethyl phosphorylcholine (MPC) has been widely used to repel protein adsorption and bacterial adhesion as an anti-biofouling material [26,27,28,29,30,31,32]. In the oral environment, microbial biofilm accumulation remains a major cause of prosthesis failure. Studies have shown that approximately more than 50% of dental restoration failures are associated with secondary caries and biofilm-induced complications [33]. Additionally, commercial DLP resins typically exhibit flexural strength values ranging from 70 to 120 MPa and limited resistance to bacterial adhesion, necessitating improved formulations for clinical use [34]. Several medical devices incorporating MPC have been reported clinically, including artificial blood vessels, implantable artificial hearts, and artificial lungs [35,36,37]. Moreover, the incorporation of MPC offers several advantages over conventional antimicrobial strategies such as quaternary ammonium salts (QASs) or nanoparticle-based fillers [38,39,40,41]. While QASs and metallic nanocomposites function via contact killing or ion release mechanisms, they are often associated with cytotoxicity, loss of activity over time, and possible bacterial resistance. In contrast, MPC achieves anti-biofouling effects through the formation of a highly hydrated zwitterionic surface that passively resists protein adsorption and bacterial adhesion without relying on chemical release [26,27,28,29]. This non-leaching, biocompatible mechanism makes MPC particularly attractive for long-term intraoral applications, where sustained performance and safety are critical. While MPC has been extensively studied as a bioinert additive in biomedical materials, its integration into photocurable resin systems suitable for high-resolution DLP printing remains largely unexplored. The present study represents the efforts to formulate a DLP-compatible MPC-containing composite with tunable viscosity, photopolymerization characteristics, and anti-biofouling function. The MPC concentration range (1–7 wt%) was selected based on preliminary solubility testing and literature reports indicating effective biofouling suppression within this range [36]. Similarly, filler loadings of 10–40 vol% were chosen to balance rheological flow, light penetration, and mechanical reinforcement within the constraints of DLP printability.

The present study aims to develop a novel anti-biofouling resin composite by incorporating MPC into a photocurable dental resin matrix. In addition, γ-MPS-treated silica-zirconia powder will be incorporated as an inorganic filler to investigate its influence on the mechanical behavior, including flexural strength and stiffness, of the composite. The resin formulation will be tailored for compatibility with digital light processing (DLP) 3D-printing technology. The effects of varying MPC contents (0–7 wt%) and filler loadings (10–40 vol%, with fixed MPC content of 7 wt%) will be systematically examined in terms of rheological behavior, photopolymerization kinetics (evaluated using Fourier-transform infrared spectroscopy, FTIR), mechanical performance, fractographic microstructural behavior, bacterial adhesion, and protein adsorption, with the goal of identifying an optimized formulation for potential dental applications.

## 2. Materials and Methods

### 2.1. Constituents of Photocurable Resin Composite Suspension

Constituents of the resin composite suspension for achieving anti-biofouling effects are summarized in Table 1. Photocurable binders, zwitterionic material, silica-zirconia powder, dispersant, and photo-initiator were used to prepare a resin composite for the DLP process. γ-MPS (γ-methacryloxypropyltrimethoxylsilane)-treated silica-zirconia powder was used to enhance chemical bonding between organic components and inorganic components. This silane coupling agent promotes covalent bonding with the methacrylate monomers and enhances interfacial adhesion.

In this work, photocurable binders having two functional acrylate groups (UDMA and TEGDMA) were used to provide mechanical strength [42], while HEMA was used as an active diluent with an ability to dissolve MPC powder [43]. As the zwitterionic material, MPC was incorporated into the resin matrix to provide anti-biofouling ability.

### 2.2. MPC-Incorporated Resin Matrix Preparation

To evaluate the solubility behavior of MPC, HEMA solutions containing various contents of MPC (10, 20, 30, 40, and 50 wt%) were prepared by dissolving MPC powder into HEMA using magnetic stirring. The solubility was roughly assessed by observing the optical appearance of each HEMA/MPC solution, such as transparency and absence of phase separation.

Based on this solubility screening, the final formulation of the resin matrix was determined to consist of 50 wt% UDMA, 20 wt% TEGDMA, and 30 wt% HEMA. Within this resin matrix, MPC was incorporated at concentrations of 0, 1, 3, 5, and 7 wt% (with respect to the total resin weight) by dissolving the powder into the HEMA phase during resin preparation, as presented in Table 2. This composition was chosen to allow for high MPC incorporation while maintaining acceptable viscosity and mechanical properties for DLP printing. Viscosities of MPC-incorporated resins were measured using a cone/plate rheometer (MCR102, Anton-Paar, Austria).

### 2.3. Resin Composite with Inorganic Filler

The compositions of resin composites are summarized in Table 3. As a photocurable binder, a resin containing 7 wt% of MPC was used in this work. Resin composites containing various amount of γ-MPS-treated silica-zirconia powder (10, 20, 30 and 40 vol%) were prepared via a vigorous mixing process with the dispersant and resin matrix using a planetary centrifugal mixer (Hantech Co, Ltd., Gyeonggi-do, Republic of Korea) for 40 min at 1000 rpm. Apparent viscosities of prepared resin composites were measured using a cone/plate rheometer.

### 2.4. DLP Process for Specimen Preparation

To examine effects of MPC content on mechanical properties and anti-biofouling abilities, various resins with various MPC contents (0, 1, 3, 5, and 7 wt%) were photocured using a commercial DLP machine (3DP-210DS; CUBICON Inc., Gyeonggi-do, Republic of Korea). The intensity, wavelength, and x-y resolution of the DLP engine were ~ 164 W/m^2^, 405–450 nm, and ~57 μm, respectively. Prior to the DLP process, 0.5 wt% of the photo-initiator was added to the resin, followed by mixing using a centrifugal mixer for 10 min at 1000 rpm. Subsequently, prepared resin matrices were photocured to produce rectangular bars for measuring flexural strength and cylinders for investigating protein-repelling and anti-biofouling ability according to a predetermined 3D design (rectangular bar: ~2.5 mm (w) × 2.0 mm (h) × 30.0 mm (l) and cylinder: 12.0 mm (d) × 2.0 mm (h)). To examine the effect of inorganic filler incorporation on resin containing 7 wt% MPC, various resin composites containing different γ-MPS-treated silica-zirconia powder contents (0, 10, 20, 30, and 40 vol%) were used to prepare rectangular bars and cylinders of the same size.

### 2.5. Photocurable Behavior Analysis

Photopolymerization behavior of MPC-incorporated resins containing various MPC contents (0, 1, 3, 5, and 7 wt%) was characterized using FT-IR. For this measurement, a 0.5 wt% of the photo-initiator was added to the resin, followed by vigorous compounding using a centrifugal mixer for 10 min. The photopolymerization behavior of the resin was monitored by decreases in two C=C double-bond regions: a 1600–1660 cm^−1^ region for C=C stretching and a 1400–1430 cm^−1^ region for C=C twisting. Unchangeable C=O region at 1750 cm^−1^ provided an internal reference for quantification of the C=C bond peak area decline [44].

(1) $$\mathrm{Percent}\mathrm{conversion}\;[\%]=\frac{{\left[\frac{{Area}_{1620}+{Area}_{1410}}{{Area}_{1750}}\right]}_{0}-{\;\left[\frac{{Area}_{1620}+{Area}_{1410}}{{Area}_{1750}}\right]}_{t}}{{\left[\frac{{Area}_{1620}+{Area}_{1410}}{{Area}_{1750}}\right]}_{0}}\;\times 100$$

### 2.6. Mechanical Properties Measurement

Mechanical properties of MPC-incorporated resin with various MPC contents (0, 1, 3, 5, and 7 wt%) (*n* = 5 for each condition) and resin composites with various inorganic filler contents (0, 10, 20, 30, and 40 vol%) (*n* = 6 for each condition) were evaluated via 3-point flexural strength tests using a screw-driven load frame (OTU-05D; Oriental TM Corp., Republic of Korea). Specimens with dimensions of ~ 2.5 mm (w) × 2.0 mm (h) × 30.0 mm (l) were tested using a span of 20 mm and a crosshead speed of 1 mm/min. The applied load and displacement were recorded during the testing process. Flexural strengths of specimens were computed by considering the load at fracture.

### 2.7. Bacterial Adhesion Analysis

*E. coli* (ATCC PTA-5073) and *S. aureus* (ATCC 6538) cultured in LB (Luria-Bertani) broth were used. For sample preparation, 1 mL of each bacterial suspension (1.3 × 10^7^ CFU/mL) was added to each disc-shaped specimen in a 24-well plate. To simulate the humid oral environment, sterile water was placed in surrounding wells and in a separate beaker inside the incubator to maintain elevated local humidity during incubation. Plates were incubated at 37 °C for 24 h under controlled ambient humidity. Specimens were gently washed twice with phosphate-buffered saline (PBS; Gibco, Waltham, MA, USA) to remove non-adherent bacteria. Attached bacteria were harvested in 1 mL LB broth by vortexing for 5 min. Then, 100 μL harvested bacterial suspension was spread onto a solid LB agar plate and incubated at 37 °C for 24 h. Total number of colonies was then counted.

### 2.8. Protein Adsorption Test

As-prepared disc-shaped specimens were immersed in fresh PBS for 24 h at 37 °C, followed by immersion in a bovine serum albumin (BSA; Difco, Franklin Lakes, NJ, USA) protein solution. To simulate the humid oral environment during incubation, sterile water was placed in the surrounding wells of the 24-well plate and in a separate beaker inside the incubator to maintain elevated local humidity. The plates were incubated at 37 °C for 4 h under controlled ambient humidity. After incubation, specimens were gently rinsed twice with fresh PBS to remove non-adherent proteins. The amount of adsorbed protein was measured using 200 μL micro-bicinchoninic aid (Thermo fisher Scientific, Waltham, MA, USA), followed by shaking for 5 min using a microplate shaker. Quantitative analysis of adsorbed proteins on the specimens’ surface was performed using a Pierce BCA Protein Assay Kit (Thermo fisher Scientific, Waltham, MA, USA). Optical density (OD) of each sample was measured with a microplate reader (Epoch; BioTek Instruments, Winooski, VT, USA) at 480 nm wavelength.

### 2.9. Statistical Analysis

Unless otherwise stated, all experiments were performed in triplicate (*n* = 3), and results are presented as mean ± standard deviation. Unless otherwise stated, all experiments were conducted under standard laboratory conditions at room temperature (25 ± 1 °C) and relative humidity of 40 ± 5%. Samples used for rheological, mechanical, and biological experiments were randomly selected from each formulation batch to minimize selection bias and ensure representative measurements. For biological assays, including protein adsorption and bacterial adhesion, five independently fabricated specimens per group were evaluated on the same day, and each test was performed three times. Unless otherwise stated, this approach was used to ensure sufficient statistical power and data reliability. All statistical analyses were carried out using one-way analysis of variance (ANOVA) with MATLAB (The MathWorks, Inc., Natick, MA, USA). A *p*-value of less than 0.05 was considered statistically significant. Differences are indicated by asterisks in corresponding figures to visually represent statistical distinctions.

## 3. Results and Discussion

### 3.1. Rheological Behavior of MPC-Incorporated Resin Matrix

**Figure 1:**
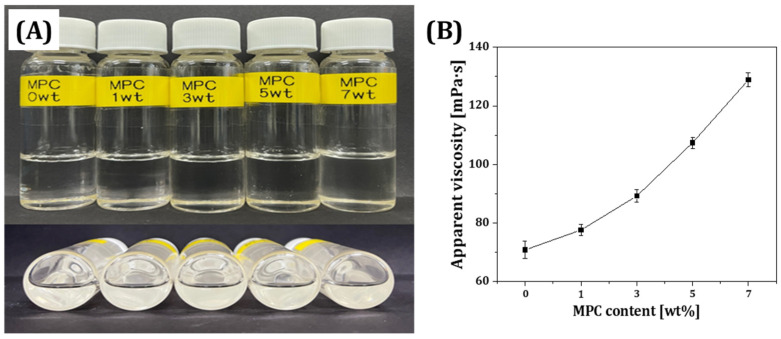
(**A**) Optical images of resins containing different MPC contents (0, 1, 3, 5, and 7 wt%) and (**B**) apparent viscosity as a function of MPC content.

The rheological behavior of the resin matrix containing various concentrations (0, 1, 3, 5, and 7 wt%) of MPC was characterized with a rheometer to determine the applicability of using the conventional DLP process. Results of the solubility of MPC in resin with various concentrations suggested that the MPC polymer was completely dissolved in resin without causing undesirable gelation or separation (Figure 1A). As the dissolved MPC content increased in the resin, the apparent viscosity was dramatically increased (Figure 1B). This finding suggested that the increase in charged molecules dissolved in polar resin led to an increase in the apparent viscosity [45,46]. As a result, the apparent viscosity of the MPC-incorporated resin matrix increased from 70.8 ± 3.0 mPa·s to 128.9 ± 2.4 mPa·s with an increase in the amount of incorporated MPC amount.

### 3.2. Photopolymerization Behavior of MPC-Incorporated Resin Matrix

**Figure 2:**
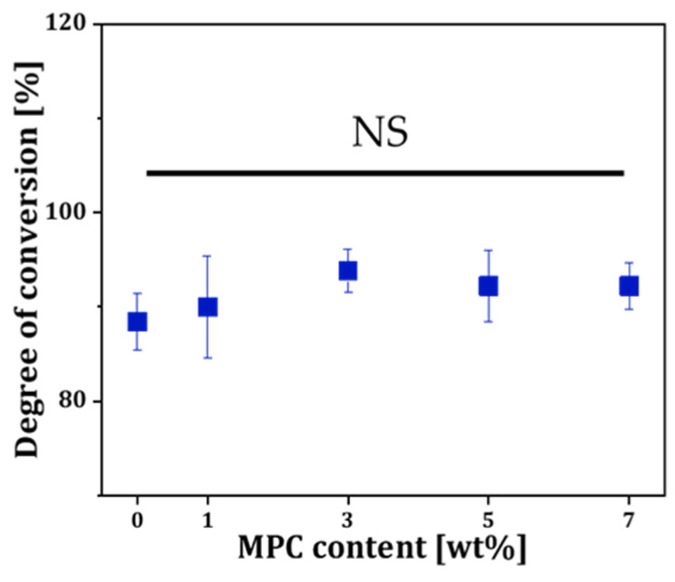
Degree of conversion of double bonds in MPC-incorporated resins.

The photopolymerization behavior of the MPC-incorporated resin depending on MPC contents was characterized by FT-IR [44]. Figure 2 displays the calculated percent of conversion from the deduction of the C=C double bond after the photocuring process considering unchangeable C=O resin as the internal reference. The percentage of conversion was slightly increased from 88.4 ± 3.0% to 92.2 ± 2.5% (Figure 2). However, due to overlapping standard deviations, the differences were not statistically significant. This result suggests that the MPC dissolved in resin did not interfere with photopolymerization behavior, indicating that the resin composite formulation remains suitable for DLP fabrication within the tested concentration range.

### 3.3. Mechanical Properties of Resin Incorporated with MPC

**Figure 3:**
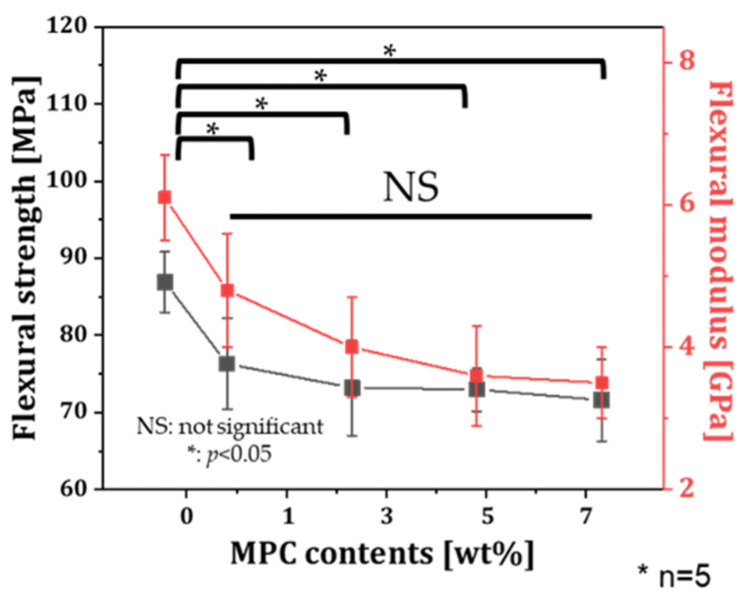
Flexural strength and flexural modulus as a function of MPC contents.

To evaluate the effect of MPC content on the flexural strength of specimens, rectangular specimens produced with various MPC contents (0, 1, 3, 5, and 7 wt%) were evaluated. The flexural strengths of specimens produced with the MPC-incorporated resin were slightly decreased with increasing MPC contents (Figure 3). This result might be due to the increase in MPC, which is inert to photopolymerization. However, note that the flexural strength of specimens with MPC incorporation regardless of the content showed no statistical difference. Specimens produced with the highest MPC content of 7 wt% showed the lowest flexural strength of 71.6 ± 5.3 MPa with a flexural modulus of 1.5 ± 0.2 GPa, which exceeded the specification requirement stated in ISO 10477 (i.e., flexural strength higher than 50 MPa) [47].

### 3.4. Bacterial Adhesion Test of Resin Incorporated with MPC

**Figure 4:**
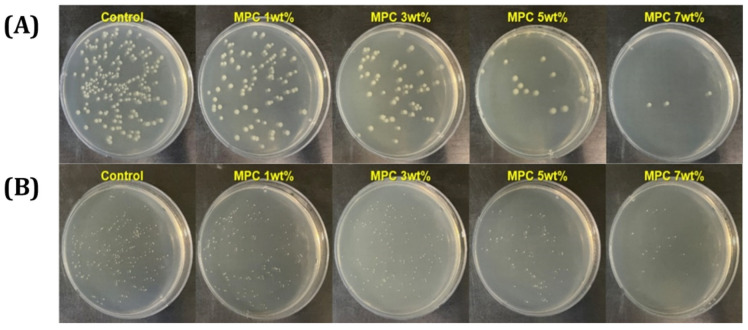
Representative optical images of CFU morphology of (**A**) *E. coli* and (**B**) *S. aureus*.

**Figure 5:**
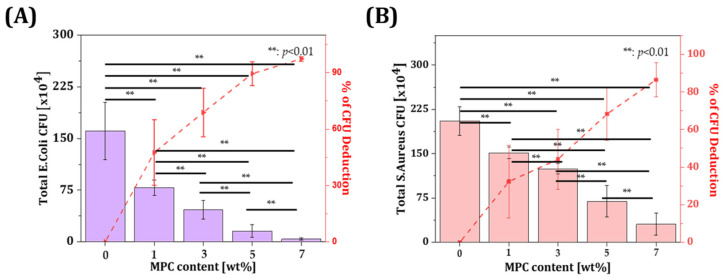
Quantitative analyses of CFU count and decreased percentage of (**A**) *E. coli* and (**B**) *S. aureus* according to MPC content.

Colony morphologies of *E. coli* and *S. aureus* on agar plates were observed. The colony forming unit gradually decreased with increasing contents of MPC incorporated in the resin compared to the control group without MPC incorporation (Figure 4). The number of CFUs in the group with MPC incorporation and the percentage of deduction compared to that in the control group was evaluated (Figure 5). The results showed that the incorporation of 7 wt% MPC resulted in 97.4 ± 1.2% deduction for the *E. coli* group and 86.5 ± 9.0% deduction for the *S. aureus* group compared to the group without MPC incorporation. These results suggest that the incorporation of MPC in the resin hindered the adsorption of bacteria on the surface of the specimen.

These findings are consistent with previous reports where MPC was shown to significantly reduce protein and bacterial adhesion. For example, a previous study demonstrated 3 wt% MPC in a BisGMA-based resin composite reduced *S. mutans* by over 90%, without compromising flexural strength [36]. Furthermore, in a multifunctional adhesive formulation, it was reported that 7.5 wt% MPC in combination with calcium phosphate fillers reduced in vitro biofilm formation by over four orders of magnitude [48]. Compared to these, our results show that even without synergistic antimicrobial additives, 7 wt% MPC alone achieved >97% reduction against *E. coli* and >86% against *S. aureus*, indicating strong anti-biofouling performance in a DLP-compatible matrix. While *E. coli* and *S. aureus* were used in this study to assess the general antibacterial performance of the resin composites, they are not representative of oral pathogens. In future studies, more clinically relevant oral bacteria such as *S. mutans* and *P. gingivalis* will be included to evaluate the material’s applicability to dental environments more accurately.

### 3.5. Protein-Repelling Ability Test

**Figure 6:**
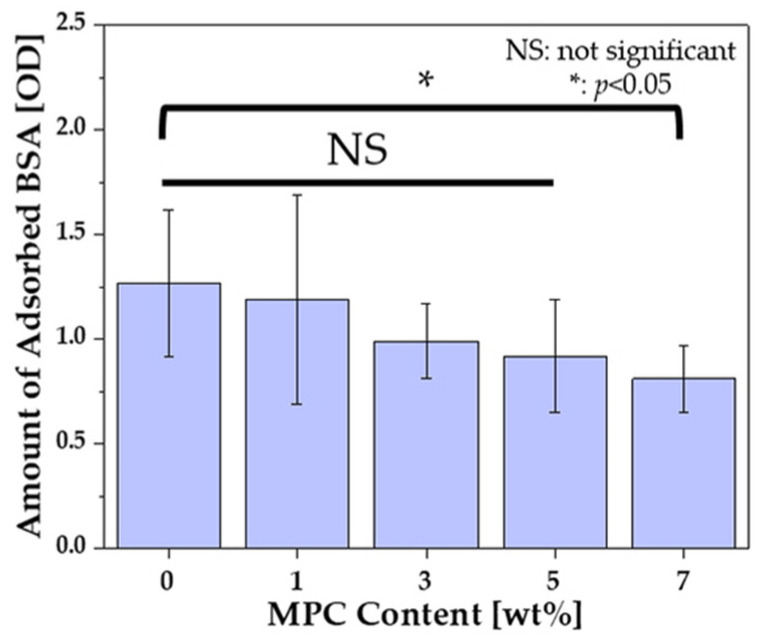
Protein-repelling ability of resin containing different MPC contents.

The amount of absorbed BSA was significantly lower in specimens produced with 7 wt% MPC than that of control. Although a decreasing trend in protein adsorption was observed with increasing MPC content, there was no statistically significant difference in the amount of adsorbed BSA among resins with different MPC contents below 7 wt%. The OD value indicated the amount of protein adsorbed on the specimen. The OD value was 1.27 ± 0.35 for the specimen produced without MPC and 0.81 ± 0.16 for the specimen produced with 7 wt%. While this difference between the control and 7 wt% MPC group was statistically significant (Figure 6), overlapping standard deviations among lower MPC groups suggest that the reductions in protein adsorption at intermediate concentrations (1–5 wt%) should be interpreted with caution. Collectively, these results highlight a mechanical–biofunctional trade-off associated with MPC incorporation. While increasing MPC content significantly reduced protein adsorption (Figure 6) and bacterial adhesion (Figure 4 and Figure 5)—with 7 wt% MPC yielding a 97.4 ± 1.2% reduction in *E. coli* and an 86.5 ± 9.0% reduction in *S. aureus*—it also resulted in a slight but consistent decrease in flexural strength (Figure 3). The flexural strength of the 7 wt% MPC group (71.6 ± 5.3 MPa) remained above the ISO 10477 threshold (50 MPa) but was, nonetheless, notably lower than that of the control. This decline may be attributed to the inert nature of MPC, which can interfere with polymer network formation. Nevertheless, the substantial gains in anti-biofouling performance outweigh the modest mechanical compromise, particularly for applications where surface hygiene is critical. Therefore, the incorporation of 7 wt% MPC represents a balanced optimization point that maximizes biological functionality while preserving acceptable mechanical integrity for intraoral applications.

### 3.6. Characterization of γ-MPS-Treated Silica-Zirconia Inorganic Filler

**Figure 7:**
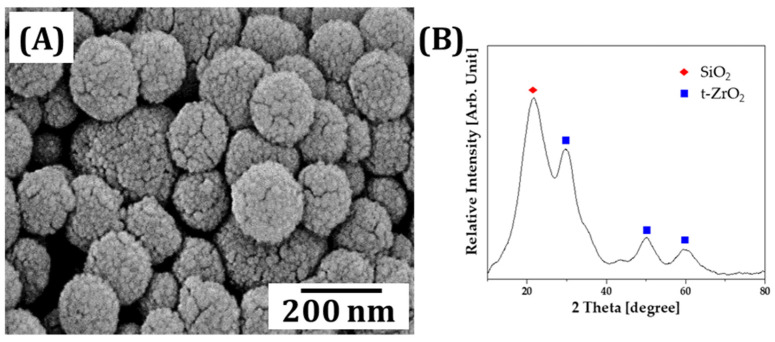
(**A**) FE-SEM image showing the morphology of γ-MPS-treated silica-zirconia powder and (**B**) XRD pattern of the powder.

Resin containing 7 wt% of MPC exhibited relatively high mechanical strength, with an excellent anti-biofouling ability (c.f. Figure 3 and Figure 5). It is known that γ-MPS-treated silica-zirconia powder, an inorganic filler, can be incorporated into a resin matrix to reinforce mechanical properties while preserving the anti-biofouling ability [49]. Furthermore, it is noteworthy to mention that γ-MPS-treated fillers form covalent bonds and establish micromechanical interlocking with the resin matrix enhancing structural integrity [50]. Meanwhile, MPC’s zwitterionic chains generate a hydrated surface layer that inhibits biofouling, while secondary interactions further stabilize the filler–resin interface [38]. A representative FE-SEM image of the powder morphology is shown in Figure 7A. An XRD pattern of the inorganic filler indicated that this powder comprises polycrystalline silica with tetragonal-phased zirconia (Figure 7B). Surface treatment of the as-prepared powder resulted in a polymer-coated surface for enhancing organic–inorganic bonding strength after photopolymerization.

### 3.7. Rheological Behavior of γ-MPS-Treated Silica-Zirconia-Filled Resin Composite

**Figure 8:**
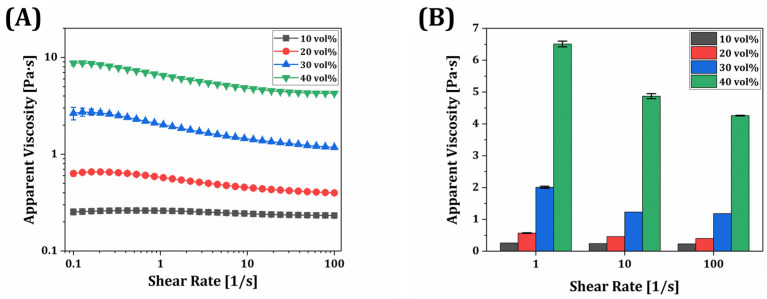
(**A**) Apparent viscosities as a function of shear rate obtained from γ-MPS-treated silica-zirconia-reinforced resin composites with various filler contents and (**B**) their apparent viscosities measured at various shear rates.

To evaluate the rheological properties of the γ-MPS-treated silica-zirconia-reinforced resin composites, the apparent viscosity as a function of the shear rate was measured for various filler contents (10, 20, 30, and 40 vol%). As shown in Figure 8A, all composites exhibited shear-thinning behavior, with viscosity decreasing as the shear rate increased from 0.1 to 100 s^−1^. It is noteworthy to mention that the 10 vol% composite showed nearly Newtonian behavior, with minimal change in viscosity across shear rates ranging from 0.1 to 100 s^−1^. In contrast, the 40 vol% composite demonstrated a marked shear-thinning profile, with viscosity decreasing by more than 50% across the same shear range. This trend indicates increased particle–matrix interaction and network formation at higher filler contents, which can be disrupted under shear, thereby reducing viscosity. This trend is typical for resin-based suspensions containing particulate fillers and is beneficial for digital light processing (DLP) printing, allowing for efficient recoating and flow during layer-by-layer fabrication [51,52].

Despite the incorporation of a relatively high filler fraction, the composite containing 40 vol% of γ-MPS-treated silica-zirconia showed a manageable viscosity profile for DLP printing. As shown in Figure 8B, at 1 s^−1^, its viscosity was 6.51 ± 0.09 Pa·s, which is substantially higher than those of the lower loading groups but still within acceptable limits for photopolymer resin recoating under moderate shear. The 10–30 vol% composites displayed significantly lower viscosities (<3 Pa·s at 1 s^−1^), ensuring good flowability. Note that the viscosity difference in each resin composite was statistically significant, which was notably increased with the filler content.

These results indicate that even at 40 vol% loading, the resin composites retain processable viscosity levels for DLP and that silane surface modification effectively mitigates viscosity escalation commonly associated with high ceramic loadings. However, the observed increase in viscosity at higher filler levels and reduced amount of photopolymer may limit light penetration and chain mobility during curing, necessitating a balance between filler content and processability.

### 3.8. Mechanical Properties of Resin Composite

**Figure 9:**
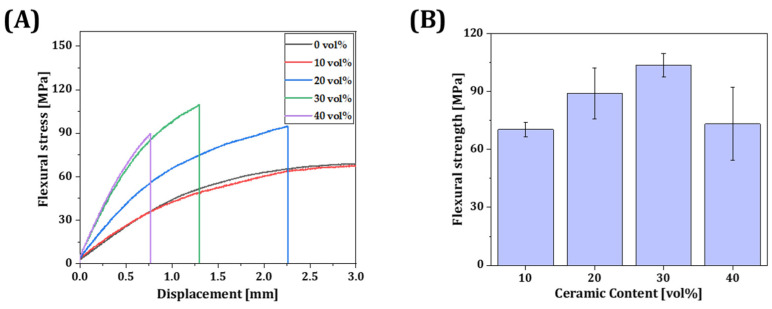
(**A**) Flexural stress as a function of displacement; (**B**) flexural strength as a function of ceramic filler content.

Flexural stress as a function of displacement was measured according to the incorporated volume of inorganic filler (0, 10, 20, 30, and 40 vol%) (Figure 9A). Specimens produced with 10 vol% γ-MPS-treated silica-zirconia powder displayed comparably long lengths of displacement until fracture occurred. On the other hand, as the incorporated volume of inorganic filler increased from 10 vol% to 30 vol%, the flexural strength and modulus dramatically increased from 70.3 ± 3.7 MPa to 103.4 ± 6.1 MPa and from 1.7 ± 0.1 GPa to 4.3 ± 0.4 GPa, respectively. However, these significantly decreased when the incorporation volume of the inorganic filler was 40 vol% (Figure 9B). By considering the decrease in flexural strength and increased viscosity of the resin composite (Figure 8), it could be stated that excessive filler loading disrupts both printability and mechanical reliability. Therefore, filler contents above 40 vol% were excluded from further investigation. Furthermore, these results suggest that the decrease in the amount of binder can diminish the adhesion region between layers through photopolymerization, which can make the strength weaker. In addition, due to an increase in the ceramic content, uneven dispersion of the powder within the resin matrix can result in a stress concentration area that can serve as initiation sites for cracks. Therefore, the resin composite containing 30 vol% exhibited the highest mechanical properties. It could be applicable for producing temporary teeth or crowns since incorporating inorganic filler can enhance mechanical properties. Although flexural strength was evaluated in accordance with ISO 10477 guidelines, where flexural properties are considered more clinically relevant for dental applications due to the predominance of bending stresses in intraoral conditions, tensile strength was not assessed in this study. Nevertheless, future work may investigate tensile properties to provide a more comprehensive understanding of the mechanical performance of the resin composite.

### 3.9. Microstructure Assessment

**Figure 10:**
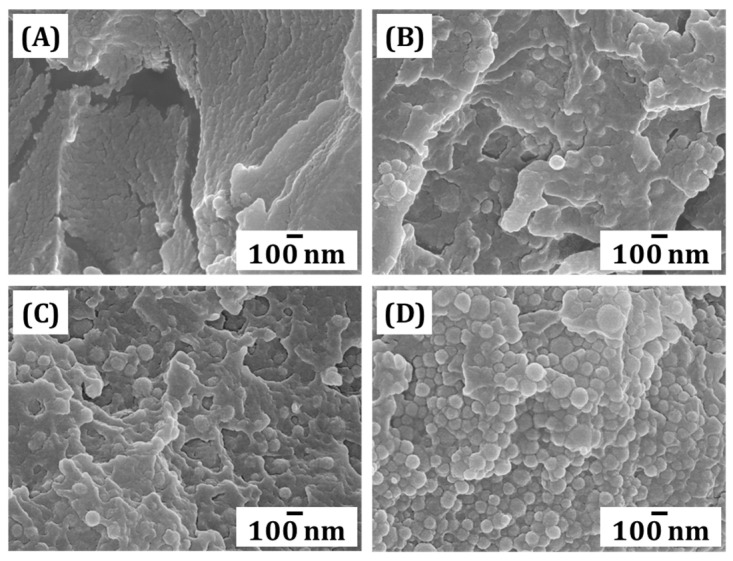
Representative FE-SEM images of fracture surfaces of (**A**) 10 vol%, (**B**) 20 vol%, (**C**) 30 vol%, and (**D**) 40 vol% of inorganic filler-incorporated resin composites.

The fractured surface of each specimen was observed using FE-SEM. Microstructures of the specimens were produced with different inorganic filler contents (10, 20, 30 and 40 vol%). The microstructures of resin composites were influenced by the content of an inorganic filler in resin composites. As the content increased, visible powders within photopolymerized frameworks increased (Figure 10). As shown in Figure 10, the filler particles are well integrated into the matrix without visible interfacial gaps, supporting effective filler–matrix bonding. This finding suggested that the inorganic powder was uniformly dispersed within the resin matrix without any indication of agglomeration. The γ-MPS-treated surface facilitated strong bonding between the inorganic filler and the organic part, resulting in an indistinguishable boundary between the powder and resin region, which was not observed at a fracture surface of the as-built green body produced with untreated powder [53]. Additionally, the improved interfacial adhesion attributed to the γ-MPS treatment likely contributed to the enhanced mechanical strength up to 30 vol% filler loading. In addition, as the content of inorganic filler increased, the portion of resin matrix significantly decreased. This result suggested that the significant reduction in mechanical strength of the 40 vol% specimen was likely attributed to the decreased portion of resin matrix, which mainly contributed to photopolymerization. While the SEM images roughly provided filler–matrix interaction, the observations were qualitative in nature. However, the absence of visible voids or agglomerates suggests good filler distribution and chemical bonding with the resin matrix. Future studies will aim to incorporate quantitative image analysis to strengthen microstructural evaluation.

### 3.10. Anti-Biofouling Ability

**Figure 11:**
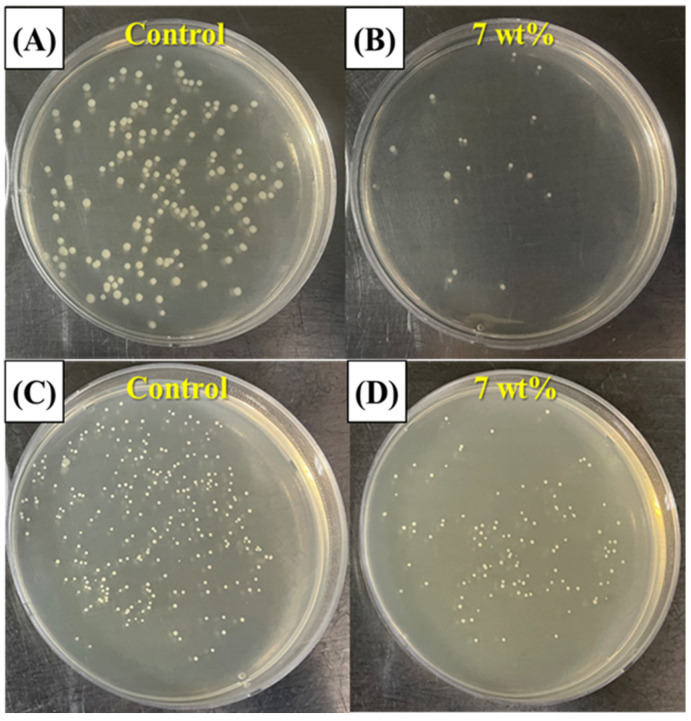
Representative image of anti-biofouling performance of MPC-incorporated resin composites assessed with CFU morphology of (**A**,**B**) *E. coli* and (**C**,**D**) *S. aureus* on surfaces of resin composites.

The anti-biofouling ability of the resin composite containing 30 vol% of inorganic filler was roughly evaluated through a bacteria adhesion test with *E. coli*, and *S. aureus*. The results are shown in Figure 11A,B for *E. coli* and Figure 11C,D for *S. aureus*. The MPC-incorporated resin composite showed reduced CFU. However, since the portion of MPC in the resin composite containing 30 vol% of inorganic filler was decreased compared to that in the resin without filler, the percentage of CFU deduction was reduced (c.f. Figure 4). In this case, an increase in the incorporated MPC content in the resin composite would be an effective way to increase anti-biofouling ability. However, the evaluation of anti-biofouling ability in this study was based on static, single-species biofilm models (*E. coli* and *S. aureus*), which do not adequately represent the complex and dynamic microbial environment of the oral cavity. Additionally, the current in vitro protocol did not incorporate thermal cycling, water sorption analysis, or long-term degradation testing, all of which are critical for assessing the clinical durability of dental materials. Therefore, future studies should adopt more physiologically relevant conditions, including multispecies biofilm models, dynamic flow systems that simulate salivary shear, and artificial aging protocols (e.g., thermocycling or water immersion) to more comprehensively evaluate both the biofunctional and mechanical performance of the developed resin composites.

## 4. Conclusions

Anti-biofouling resin composites incorporated with MPC and γ-MPS-treated silica-zirconia inorganic filler for clinical uses could be fabricated using the DLP process. The MPC content was carefully measured considering the polymerization behavior and rheology behavior of the resin composite. The anti-biofouling resin showed the highest anti-biofouling abilities when the MPC content was 7 wt%, which decreased protein adhesion effectively (decrease: 97.4% for *E. coli* and 86.5% for *S. aureus*), which showed reasonably comparable results when compared to previously studied antifouling dental composites that incorporate quaternary ammonium salts (QASs) or silver-based nanoparticles. In addition, the resin composite showed the highest flexural strength (103.4 ± 6.1 MPa) and modulus (4.3 ± 0.4 GPa) when the γ-MPS-treated silica-zirconia filler content was 30 vol%. Such optimized composition of the anti-biofouling resin composite can be adopted for dental resin applications using the DLP process and utilized in restorative dental fields where prosthetic materials should offer anti-biofouling effects. Future studies will further investigate the long-term durability of the anti-biofouling effect, cytocompatibility, and in vitro biofilm resistance using oral pathogens such as *S. mutans* and *P. gingivalis*. Furthermore, the elaboration of the understanding of mechanical properties by performing tensile tests will be conducted. Additionally, the application of the optimized resin to complex DLP-fabricated dental structures will be explored.

## Figures and Tables

**Table 1:** Constituents of the resin composite for DLP process.

Role	Inorganic Filler	Photocurable Binders		
Material	γ-MPS treated Silica-Zirconia	Urethane Dimethacrylate (UDMA)	Triethylene Glycol Dimethacrylate (TEGDMA)	2-Hydroxyethyl Methacrylate (HEMA)
Supplier	Sukgyung AT (Gyeonggi-do, Republic of Korea)	Sigma Aldrich (St. Louis, MO, USA)	TCL chemical (Portland, OR, USA)	Sigma Aldrich (St. Louis, MO, USA)
**Role**	Zwitterionic material	Dispersant	Photo-initiator	
Material	2-Methacryloyloxyethyl Phosphorylcholine (MPC)	BYK-2001	Diphenyl(2,4,6-trimethylbenzoyl) Phosphine oxide (TPO)	
Supplier	KCI Limited (Seoul, Republic of Korea)	BYK-Chemie (GmbH, Wesel, Germany)	Sigma Aldrich (St. Louis, MO, USA)	

**Table 2:** Components used to prepare MPC incorporated resin matrix for DLP printing, their amount (g) and weight fractions (wt%) calculated by considering the total amount of resin matrix. Photocurable binder: mixture of UDMA, TEGDMA, and HEMA with a weight ratio of 5:2:3.

Role	Photocurable Binders	Zwitterionic Material	Photo-Initiator
Material	UDMA	MPC	TPO
	TEGDMA		
	HEMA		
Amount [g]	20.0	0	0.1
	19.8	0.2	0.099
	19.4	0.6	0.097
	19.0	1.0	0.095
	18.6	1.4	0.093
Weight fraction [wt%]	99.5	0	0.5
	98.5	1.0	0.5
	96.5	3.0	0.5
	94.5	5.0	0.5
	92.5	7.0	0.5

**Table 3:** Components used to prepare resin composite for DLP printing, their amount (g) and weight fractions (wt%) calculated by considering the total amount of resin composite.

Role	Photocurable Binders	Zwitterionic Material	Inorganic Filler	Dispersant	Photo Initiator
Material	UDMA	MPC	γ-MPS treated Silica-Zirconia	BYK-2001	TPO
	TEGDMA				
	HEMA				
Amount [g]	18.6	1.4	5.4	0.2	0.093
			12.4	0.4	
			21.5	0.6	
			33.8	1.0	
Weight fraction [wt%]	72.4	5.4	21.0	0.8	0.4
	56.5	4.3	37.7	1.2	0.3
	44.1	3.3	51.0	1.4	0.2
	33.9	2.6	61.6	1.8	0.2

## Data Availability

The original contributions presented in this study are included in the article. Further inquiries can be directed to the corresponding author.
